# Surveillance of Hepatitis E Virus Contamination in Shellfish in China

**DOI:** 10.3390/ijerph120202026

**Published:** 2015-02-11

**Authors:** Shenyang Gao, Dandan Li, Enhui Zha, Tiezhong Zhou, Shen Wang, Xiqing Yue

**Affiliations:** 1 Department of Food Science, Shenyang Agricultral University, Shenyang 110161, China; E-Mails: gordon402@163.com (S.G.); ztz1818@163.com (T.Z.); wangshenln@163.com (S.W.); 2 Department of Animal Husbandry & Veterinary Medicine, Liaoning Medical University, No. 5-48 Renmin Street, Jinzhou 121001, China; 3 Animal Quarantine Lab, Inspection & Quarantine Technology Center of Hainan Entry-Exit Inspection & Quarantine Bureau, Haikou 570000, China; E-Mail: bioluck@126.com; 4 Department of Food Science, Liaoning Medical University, No. 5-48 Renmin Street, Jinzhou 121001, China; E-Mail: michelle_enhui@126.com

**Keywords:** hepatitis E virus, shellfish, sub-genotype 4, Bohai Gulf, RT-PCR

## Abstract

*Background*: Hepatitis E virus (HEV) has been confirmed to be a zoonotic virus of worldwide distribution. HEV contamination in the water environment has not been well examined in China. The objective of this study was to evaluate HEV contamination in shellfish in a coastal area of China. Such contamination would be significant for evaluating public health risks. *Method*s: samples of three species shellfish were collected from thirteen points of estuarine tidal flats around the Bohai Gulf and screened for HEV RNA using an in-house nested RT-PCR assay. The detected HEV-positive samples were further verified by gene cloning and sequencing analysis. *Results*: the overall HEV-positive detection rate is approximately 17.5% per kilogram of shellfish.  HEV was more common among *S. subcrenata* (28.2%), followed by *A. granosa* (14.3%) and *R. philippinarum* (11.5%). The phylogenetic analysis of the 13 HEV strains detected revealed that gene fragments fell into two known 4 sub-genotypes (4b/4d) groups and another unknown group. *Conclusions*: 13 different sub-genotype 4 HEVs were found in contaminated shellfish in the Bohai Gulf rim. The findings suggest that a health risk may exist for users of waters in the Bonhai area and to consumers of shellfish.  Further research is needed to assess the sources and infectivity of HEV in these settings, and to evaluate additional shellfish harvesting areas.

## 1. Introduction

Hepatitis E virus (HEV) is transmitted via the fecal-oral route and causes large waterborne outbreaks worldwide [1]. The zoonosis of HEV has been confirmed based on the recent reports of swine, wild boars, and wild deer serving as the reservoir for human infections [2,3,4,5,6]. HEV is a single-stranded, positive-sense, non-enveloped RNA virus that is classified in the family Hepeviridae with at least four major genotypes (G1-4). G1 and 2 only affect humans, whereas G3 and 4 are zoonotic worldwide [7]. Hepatitis E infection is endemic in areas of China. The prevalence of the infection in swine and the human HEV molecular epidemiology and phylogeny in inland China have recently been described [8], whereas, no evidence of HEV-contaminated coastal waters has been reported until now. HEV has been documented as a causative agent for food-borne illness associated with consumption of contaminated shellfish [9,10,11], therefore, it seems reasonable to speculate that shellfish, as an ideal natural indicator, might reflect the presence of HEV circulation in environmental waters. This study sought to survey HEV contamination in edible shellfish collected from estuarine tidal flats along the Bohai Gulf in China, which with over 230 million residents, is one of the most densely populated urban agglomeration areas in the country.

## 2. Experimental Section

### 2.1. Shellfish Sampling and Processing

**Figure 1:**
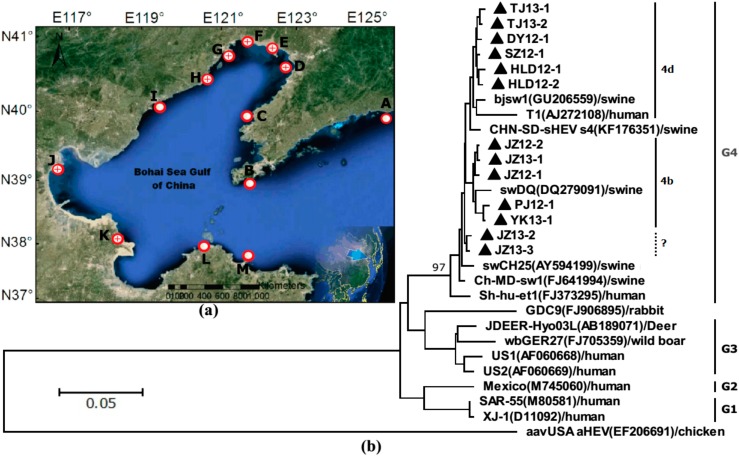
Locations of shellfish sampling and phylogenetic trees of hepatitis E virus. (**a**) Locations of shellfish collected in the Bohai gulf rim. The site of HEV RNA positive detection is marked by a positive sign within red circle 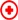 and that of negative detection’s with a only a red circle 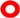. (**b**) Phylogenetic trees of hepatitis E virus (HEV) were constructed based on partial genomes. Each partial ORF3 (287basepairs) of 13 different clones of JZ12-1, JZ12-2, HLD12-1, HLD12-2, PJ12-1, DY12-1, SZ12-1, JZ13-1, JZ13-2, TJ13-1, TJ13-2, YK13-1and JZ13-3 (with GenBank accession No. KJ816338, KJ816339, KJ816336, KJ816337, KJ816343, KJ816335, KJ816344, KJ816340, KJ816341, KJ816345, KJ816346, KJ816347 and KJ816342, respectively.) was analyzed by the neighbor-joining method. The bootstrap value correspond to 1,000 replications of avian HEV was used as an outgroup. All nucleotide sequences determined in this study were marked by ▲. Other HEV sequences were retrived from GenBank.

A total of approximate 126 kg of shellfish samples consisting of 35 kg *A. granosa*, 39 kg *S. subcrenata* and 52 kg *R. philippinarum* were collected from thirteen estuarine tidal flats along the Bohai Gulf in China from March 2012 to December 2013 (see Figure 1 and Table 1). Considering the weight differences between individual samples, the contamination rate of HEV within each shellfish species was examined and calculated as a percentage of shellfish weight per kilogram instead of by individual analysis. The 126 kg of raw shellfish samples were divided into three groups by species and 1 kg of each shellfish species was regarded as a tested sub-sample which approximately contains 66–100 individual shellfish. Therefore, there were 126 sub-samples overall, consisting of 35 *A. granosa*, 39 *S. subcrenata* and 52 *R. philippinarum* sub-samples.

Each sub-sample was pretreated as previously described with slight modifications [12]. Briefly, the digestive tissues dissected from each sub-sample of shellfish were composited together. After weighing, a 30 ± 5 g tissue sub-sample was transferred to a 500 mL centrifuge bottle and diluted (1:7 wt/vol) with 210 ± 35 mL tryptose phosphate broth (contains 10% tryptose, 0.05 M glycine at pH 9.0) then homogenized by shaking at 250 rpm for 30 min in a Vortex Mixer (IKA Werke GmbH & Co., Staufen, Germany). The emulsion formed was equally distributed into 4–6 Corning 50 mL tubes and centrifuged at 10,000× g for 30 min at 4 °C; The supernatant in each tube was collected for pH adjustment to 7.0 before weighing the volume, then polyethylene glycol (PEG) 6000 MW (Sigma Chemical Co., St. Louis, MO, USA) and NaCl were added to final concentrations of 8% and 0.7% (wt/vol), respectively. The subsequent approximately 200 ± 20 mL mixed liquid was precipitated at 4 °C overnight and thereafter centrifuged at 10,000× g for 30 min at 4 °C. The resulting pellet was resuspended in 10–15 mL of PBS for further concentration by ultrafiltration using Amicon Ultra-15 Centrifugal Filter Units (Millipore, Billerica, MA, USA). After centrifugation at 4000× g for 45 min, the final volume of 200 μL concentrated sub-sample was stored at ‒80 °C until use.

**Table 1:** Statistics of HEV-positive detection rate by percentage per kg of each species of shellfish.

Location A–M	Kg of Shellfish Collected, by Species			HEV RNA Positive ^R^	Final verified ^S^
	*A. granosa*	*S. Subcrenata*	*R. philippinarum*		
A: Dandong	NA	2	3	0	
B: Dalian	2	3	4	0	
C: Wafangdian	NA	3	3	0	
D: Yingkou	3	5	4	1	1
E: Panjin	3	3	4	4	1
F: Jinzhou	8	6	9	7	5
G: Huludao	5	3	3	4	2
H: Suizhong	4	2	2	1	2
I: Qinhuangdao	NA	2	2	0	1
J: Tianjin	5	3	6	4	1
K: Dongying	3	4	5	1	
L: Penglai	NA	3	4	0	
M: Yantai	2	NA	3	0	
Total ( detection rate C )	35 (5/35, 14.3%)	39 (11/39, 28.2%)	52 (6/52, 11.5%)	22 (5 + 11 + 6) (22/126, 17.5%)	13

Notes: NA, no available sufficient samples; numbers with double underscores indicate a HEV-positive sample was detected in the species group; ^R^, HEV-positive results were screened out by a self-designed nRT-PCR method and “(5 + 11 + 6)” represents the number of HEV-positive detections from each species group of *A. granosa, S. subcrenata and R.philippinarum*, respectively; ^S^, HEV-positive results were further verified by gene cloning and sequencing analysis and the repetitive positive sequences were excluded. The final submitted HEV-positive sequences were assigned accession in GenBank as listed in Table 2; ^C^, The detection rate of each species was calculated by (HEV-positive number of each species/total species group weight (kg)) × 100%” and the total detection rate was calculated by (total detected HEV-positive numbers/total sample weight of shellfish) × 100%” namely, (22/126) × 100% = 17.5% per kg. Locations A-M (a total of 13 sampling points) are specifically marked in the map of Figure 1a; Species of shellfish was named according to en.wikipedia.org.

### 2.2. HEV Screening and Phylogenetic and Evolutionary Analysis

The viral RNA was extracted from each 200 μL concentrated sub-sample using the QIAmp viral RNA mini kit (Qiagen, Hilden, Germany). The presence of target HEV RNA was detected by a self-designed nested RT-PCR (nRT-PCR). First round amplification launched with external forward primer HEV-F1 (5'-CGGATGGAATGAATAACATGT-3') and external reverse primer HEV-R1 (5'-CACGTGAATCAACATCAGG-3'), which correspond to the nucleotide residue 5133–5163 and 5549–5567 regions of the G4 strain swDQ (DQ279091), respectively. Each cycle consisted of denaturation at 95 °C for 30 s, primer annealing at 55 °C for 30 s, and extension at 72 °C for 60 s, followed by final extension at 72 °C for 10 min. Two microliters of the first round PCR product was used as template for a nested PCR with internal forward primer HEV-F2 (5'-CCTATGCTGCCC GCGCCACCG-3'; nucleotide residues 5225–5245) and internal reverse primer HEV-R2 (5'-AACG GCGAAGCCCCAGCT-3', nucleotide residues 5494–5511) under the same conditions. In order to exclude contamination of samples in the laboratory, blank controls (n = 5) were run along with samples. The final 287 bp nRT-PCR products were purified using the QIAquick PCR purification kit (Qiagen) and cloned into TA cloning vector pMD18-T (Takara, Japan). Each of the 20 clones were sequenced by Shanghai Sagon Bioengineering Co. Ltd. (Shanghai, China). After sequencing analysis, the verified original sequences were submitted to GenBank.

Phylogenetic trees were constructed using the neighbour-joining method by MEGA software version 6.0 and the reliability of the clusters were assessed by bootstrapping using 1000 replicates. The analyses were carried out based on a highly conservative overlapping region of ORF2 and 3 of HEV (in fact, ORF3 was totally included in overlapping region). To obtain the high accurate and conclusive results, thirteen isolated gene fragments of HEV were analyzed and compared to the most representative reported animal and human HEV G1-4 and some major endemic sub-genotypes (4b and 4d) which were retrieved from GenBank.

## 3. Results and Discussion

**Figure A1:**
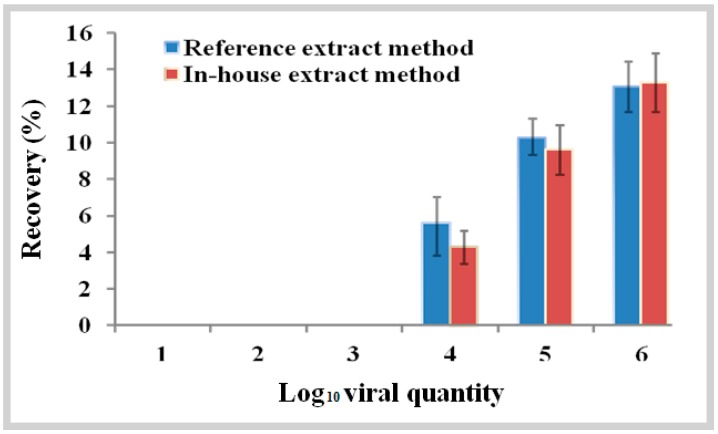
Comparison of two methods for the recovery of HEV inoculated into shellfish digestive tissue using qRT-PCR. Recoveries with the in-house method in comparison with the reference method at gradient initial viral quantity in 10 mL supernatant samples of shellfish digestive tissue. Recoveries by reference method with the same gradient initial viral quantity in individual shellfish digestive tissue directly. The detailed procedure of the reference method (based on reports by Jothikumar *et al*. in 2006 [21,22]) with some slight modifications conducted in this test is as follows: Step 1. To prepare the test initial virus sample by qRT-PCR. The stored original previous HEV-positive supernatant detected by RT-PCR was further quantified by reference qRT-PCR assay [22]. The virus supernatant was diluted by gradient of n × Log10 (n = 1, 2, 3, 4, 5 and 6), respectively, as an initial virus sample for the test. Step 2. To prepare HEV contaminated shellfish samples in a similar way. The frozen shellfish were removed for thawing 20–40 min on ice. The individual shells were opened one by one with a sterile shucking knife, ensuring that the hand holding the animal is protected with a heavy-duty safety glove. The digestive glands were dissected from the animals using scissors and forceps and transfered to a clean Eppendorf tube. The weight of the individual gland mass (2.0 ± 0.2) g was made up by adding sterile water. Step 3. The digestive gland is finely chopped up with an equivalent homogenizer to a paste-like consistency. Step 4. Add (2.0 ± 0.2) mL of proteinase K solution and mix. Incubate at (37 ± 1.0) °C with shaking at approximately 320 min^−1^ in a shaking incubator for (60 ± 5) min. Step 5. Carry out a secondary incubation by placing the tube in a water bath at (60 ± 2.0) °C for (15 ± 1) min. Step 6. Centrifuge at 3000× g for (5.0 ± 0.5) min, decant the supernatant into a clean tube, measure and record the volume of supernatant, in milliliters, and retain for RNA extraction. As shown in Figure A3, samples without results mean that the obtained Ct values were below the limitation of quantitation of the qRT-PCR test, and recoveries could not be calculated. The reference viral extract method was conducted according to ISO/TS 15216–2:2013 procedure with some slight modifications and the screening qRT-PCR assay. The results indicated that the efficiency of in-house virus extract method was comparable to that of reference method, but both of them had very low efficiency.

**Figure A2:**
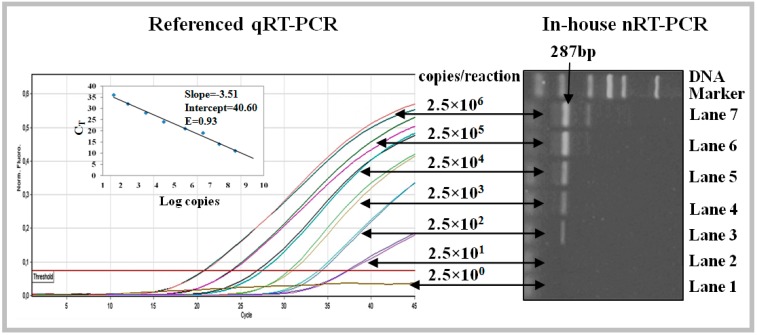
Comparison of the sensitivity of the in-house nRT-PCR and reference qRT-PCR methods for the detection of HEV RNA. The detection limit (2.5 × 10^2^ copies/reaction) of the in-house nRT-PCR was less sensitive (10-fold) than that of reference qRT-PCR [21]. This results reflected that the real presence of HEV in shellfish might be higher than the results described in this study.

**Figure A3:**
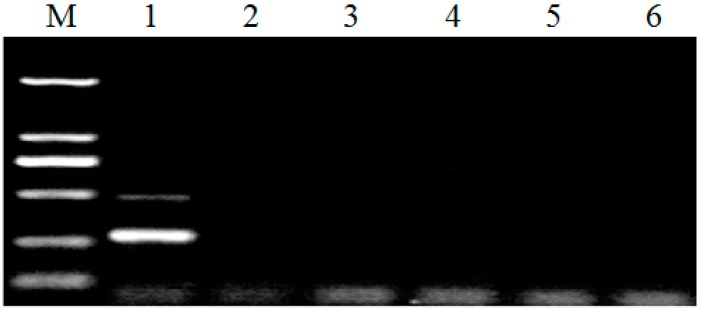
Specificity analysis of in-house nRT-PCR. M: DNA marker 2000 bp; 1: HEV positive control; 2: ddH_2_O as negative control; 3–6 are porcine reproductive and respiratory syndrome virus, hepatitis A virus, Norovirus and porcine epidemic diarrhea virus, respectively. This result indicated that the detection specificity of in-house nRT-PCR was acceptable, though the number of trials in current system is limited.

After screening by nRT-PCR, a total of 22 HEV-positive sub-samples and 104 HEV-negative sub-samples were detected out from 126 kg of shellfish, while all the blank control samples in the nRT-PCR tests were HEV-negative. The overall HEV-positive detection rate is thus approximately 17.5% per kilogram of shellfish. Among them *S. subcrenata* reached the highest HEV-positive detection rate of 28.2% per kilogram when compared with 14.3% for *A. granosa* and 11.5% of *R. philippinarum*. Given the usually extremely low virus extraction efficiencies (see Figure A1, Figure A2 and Figure A3), it was reasonable to assume that the realistic prevalence of HEV in shellfish might be much higher than the present described in this study. In the thirteen the survey sites (A–M, see Figure 1 and Table 1), there were seven sites (D–H, J and K, see Figure 1a) where HEV RNA were detectable in the local shellfish samples. All three species shellfish collected from the Jinzhou site (E, Figure 1 and Table 1) were HEV RNA detectable. The total of 22 HEV-positive samples were further verified by gene sequencing analysis. Finally, there were 13 isolated sequences found to be new HEV nucleotides corresponding to region 5225–5511 nt in the ORF3 of the reference swDQ (DQ279091) strain (see Table 2) after ruling out repetitive positive sequences. Based on this ORF3 region, phylogenetic analysis results indicated that all 13 strains sequences belonged to G4 HEV, which separately falls into two sub-genotypes 4b and 4d cluster of G4 with nucleotides sequence identities of 97.0%–99.7% and 94.6%–99.3%, respectively (see Figure 1b). In cluster 4b, the sequences of five strains isolated from sampling D, E and F sites were close to that of known sub-genotype HEV strain swDQ279091/swine/China with nucleotide sequence identities of 97.6%–98.0%. In cluster 4d, the sequences of six strains isolated from sampling G, H, J and K sites were close to that of known sub-genotype HEV strain bjsw1 GU206559/swine/China and T1 AJ272108/human/China with nucleotide sequence identities of 94.6%–98.3%. The sequences of JZ13-2 and JZ13-3 strains seem not belong to any known sub-genotype but shares identities of 98.0% with known swCH25 AY594199/swine/China.

**Table 2:** List of thirteen identified HEV RNA isolates from different species shellfish.

Collection-Dates	Isolation-Sources	Isolates-Tentative Names	GenBank-Accession Numbers
22/Mar/12	*S. Subcrenata*	JZ12-1	KJ816338
28/Mar/12	*S. Subcrenata*	JZ12-2	KJ816339
6/Apr/12	*S. Subcrenata*	HLD12-1	KJ816336
6/Apr/12	*S. Subcrenata*	HLD12-2	KJ816337
2/May/12	*R. philippinarum*	PJ12-1	KJ816343
3/May/12	*R. philippinarum*	DY12-1	KJ816335
4/Nov/12	*A. granosa*	SZ12-1	KJ816344
11/Mar/13	*S. Subcrenata*	JZ13-1	KJ816340
16/Apr/13	*A. granosa*	JZ13-2	KJ816341
25/Apr/13	*A. granosa*	TJ13-1	KJ816345
25/Apr/13	*S. Subcrenata*	TJ13-2	KJ816346
16/Nov/13	*S. Subcrenata*	YK13-1	KJ816347
19/Dec/13	*R. philippinarum*	JZ13-3	KJ816342

The risk factors for hepatitis E are related to poor sanitation in large areas of the world, and the ingestion of raw or undercooked shellfish has also been identified as the source of sporadic cases in endemic areas [13,14,15]. In this study, we examined a total of 126 kg shellfish from 13 estuary tidal locations in the Bohai Gulf in China from March 2012 to December 2013. The shellfish samples collected from seven of the 13 coastal sampling sites were detected positive for HEV RNA, and the three species of shellfish investigated, *A. granosa*, *S. subcrenata* and *R. philippinarum*, were all found to be contaminated, indicating that there might exist HEV contamination in the estuary coastal waters of China. Subsequent sequencing and phylogenetic analysis results revealed that all 13 of the isolated HEV gene sequences belonged to G4 HEV, and five isolated among them could be classified into sub-genotype 4b of human or swine HEV, while six of them belonged to 4d, and 2 of them were unknown. This can support the belief that there exist multiple HEV strains in circulation in the Bohai rim. Whether shellfish might be exposed to human or animal fecal-polluted surface runoff from coastal areas needs to be further investigated.

**Figure A4:**
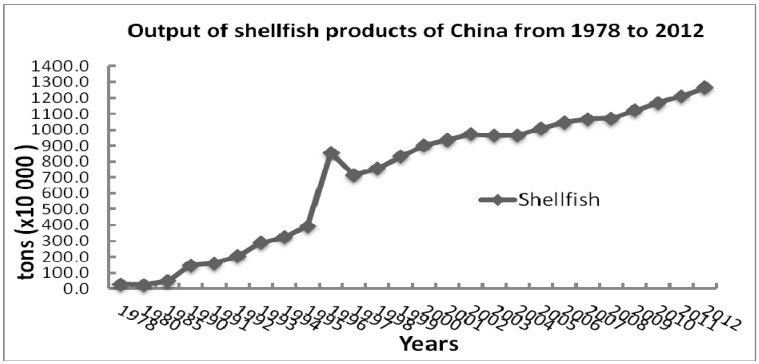
Output of shellfish products of China from 1978 to 2012. Source: China Statistical Yearbook 2013 (http://www.stats.gov.cn/tjsj/ndsj/2013/html/Z1320e.htm).

**Figure A5:**
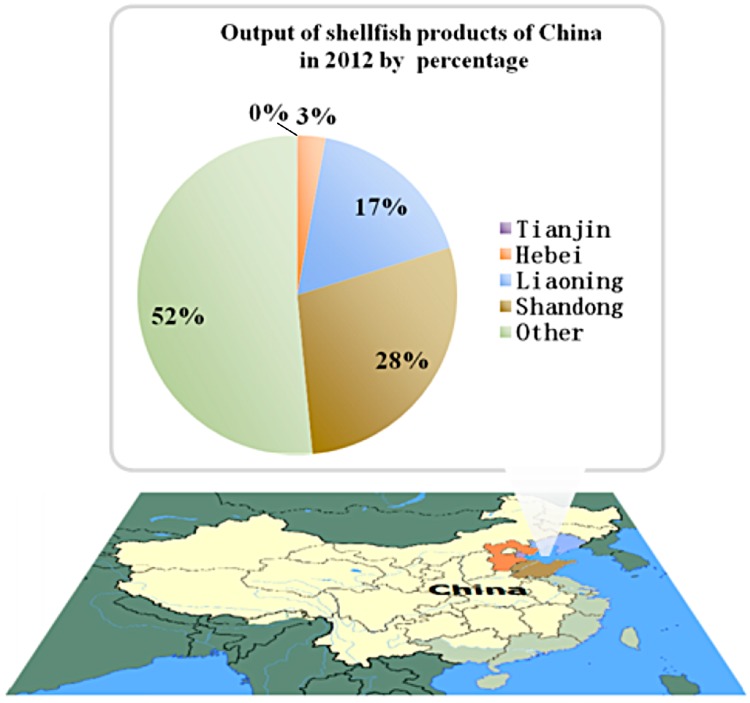
Output of shellfish products of China in 2012 by percentage. Source: China Statistical Yearbook 2013 (http://www.stats.gov.cn/tjsj/ndsj/2013/html/Z1320e.htm).

Similar surveys on the presence of HEV (genotype 3) in bivalve mollusks have been reported previously in Japan, Thailand and United Kingdom since 2007 [16,17,18]. A similar phenomenon of multiple HEV strains accumulated in the digestive tissue of shellfish was also found in the present study, for example, isolates JZ13-1, 2 and 3 demonstrated 97.0%–99.7% sequence similarity to G4 HEV, even though they were derived from the same batch of shellfish samples. In addition, the isolates JZ12-1, 2 and JZ13-1, 2, 3 detected from the same site at one year intervals were found to display 97.6%–99.7% sequence similarity. This finding suggested that the number of sub-genotypes might expand progressively and reflect HEV-contamination across geographical areas in the river upstream. Since 2000, the dominant genotype of HEV in China has been confirmed to be G4 from previous G1, though G3 HEV has recently been isolated [19,20]. The concrete reason behind this shift towards G4 HEV is unknown, but based on our survey results, the rapid growth of shellfish farming and consumption might contribute to an increasing risk of zoonotic food-borne G4 HEV circulation in coastal areas in China over the past ten years (see Figure A4 and Figure A5).

## 4. Conclusions

Thirteen new strains of gene fragments belonging to G4 HEV were detected in shellfish in China. These shellfish-source isolated HEV gene sequences could be clustered into two known sub-genotypes, 4b, 4d, and into an unknown group. This evidence indicates that there exist multiple HEV strains contaminating and circulating in the estuary water environment around the Bohai rim in China. Future research should address the possible sources of HEV in coastal areas where HEV is present in shellfish. These findings demonstrate a need for evaluating the infectivity of HEV in these settings and potential human health risks of consuming shellfish from areas where infectious HEV is present.
